# Lepromatous leprosy in Bronze Age Oman: micro-CT provides tools for paleopathology in fragmentary and commingled assemblages

**DOI:** 10.3389/fmed.2025.1521515

**Published:** 2025-03-31

**Authors:** Gwen Robbins Schug, Sangeeta Mahajan, Savannah Carter, Alexandra Leach, Kimberly D. Williams, Khaled A. Douglas, Nasser S. Al-Jahwari

**Affiliations:** ^1^Human Diversity Lab, Department of Biology, University of North Carolina Greensboro, Greensboro, NC, United States; ^2^Department of Biology, University of North Carolina Chapel Hill, Chapel Hill, NC, United States; ^3^Department of Anthropology, Temple University, Philadelphia, PA, United States; ^4^Department of Archaeology, Sultan Qaboos University, Muscat, Seeb, Oman; ^5^Department of Sustainable Tourism, Hashemite University, Zarqa, Jordan

**Keywords:** paleoradiology, micro-CT, leprosy, Bronze Age Oman, paleopathology

## Abstract

Paleoradiology uses CT scanning, digital radiography, and 3D imaging to non-invasively characterize the lives and the experience of health and disease for past people. This paper presents an analysis of micro-CT scans of leprosy in three archaeological maxillae from Dahwa, Oman (2500-2000 BCE) to characterize the natural history of disease progression on an ultra-structural level and address environmental and social factors that shaped the experience of health in Arabia during the Bronze Age. The human skeletal remains from Bronze Age Oman present a challenge for paleopathological analysis because they are fragmentary and commingled. We demonstrate microstructural characteristics of lesions in the ventral maxilla and palate that are strongly consistent with a diagnosis of lepromatous leprosy (e.g., atrophy of the anterior nasal spine, resorption of the medial alveolar process, deterioration of the piriform aperture margin, and atrophy of the nasal septum) and the utility of micro-CT for non-invasively characterizing pathology in isolated maxilla from fragmentary, commingled archaeological contexts. The presence of disfigurement, probably resulting from leprosy, in this community provides new evidence about the early migrations of pathogens responsible for leprosy, which despite being an ancient disease is still relatively poorly understood.

## 1 Introduction

Leprosy, or Hansen’s disease, is caused by infection with *Mycobacterium leprae* or *M. lepromatosis*, an intracellular parasite that affects the skin, peripheral nerves, bones, endothelial cells, mucous membranes, and eyes (1). Four million people currently live with leprosy and 200,000 new cases are diagnosed each year, primarily in India, Indonesia, and Brazil (2). It is considered a neglected tropical disease although with rising global importance as a potentially reemerging infection (3–5). Perhaps because of our long co-evolution with the pathogen, most humans have innate immunity. Estimates suggest only 5% of people exposed to *M. leprae* will become infected and of those, only 20% will contract the disease (6). Leprosy requires an extensive period of close contact for transmission and the latent period can be decades long, making diagnosis difficult. Leprosy can have disfiguring consequences if it goes undiagnosed.

The pathogens responsible for leprosy have been affecting human populations for millennia (7). The origin, early migrations, and evolution of these mycobacteria are not well understood. *M. leprae’s* lineage split from an ancestral *Mycobacterium tuberculosis* Complex (MTBC) ancestor during the Late Pleistocene and subsequently, the pathogen’s evolution was shaped by human migration patterns and trade (8). Phylogenetic and divergence timing analysis suggest contemporary strains may have emerged as early as 4500 years ago (9), perhaps with migration events associated with the Bronze Age Middle Asian Interaction Sphere (10). Some support for this hypothesis is found in the earliest published skeletal evidence for the disease from Balathal, India c. 4500–4000 BP and from three mortuary contexts at Harappa in Pakistan c. 4150–3900 BCE (11, 12). The pathogen later went through at least two population expansions beginning around 250 CE and 1600 CE, with Roman imperialism and settler colonialism transporting the disease to Europe and the Americas (13).

This paper presents paleopathological evidence for lesions strongly consistent with a diagnosis of leprosy in a highly fragmentary, commingled mortuary assemblage from Bronze Age Oman. We used micro-CT to non-invasively diagnose leprosy in three maxillae, one of which was excavated from a Bronze Age tomb and two of which were excavated from an associated bone pit at the site of Dahwa on the Batinah Coast (14–16). This is the first report of Bronze Age (2500–2000 BCE) leprosy in a community outside of South Asia that was clearly involved in trade as part of the Middle Asian Interaction Sphere. This is also the first published micro-CT characterization of lesions associated with lepromatous leprosy in isolated archaeological maxilla fragments, demonstrating the utility of this method for paleopathological analysis of archaeological skeletons from highly fragmented, disassociated, and commingled contexts.

This paper also suggests morphological changes to the ventral maxilla and palate may be useful for clinical diagnostics using CT-scanning in diagnostically-challenging cases of leprosy. In living patients, 70% of leprosy cases are diagnosed through microscopic detection of the bacilli, peripheral nerve thickening, and loss of sensation in hypopigmented or reddened skin patches (2, 17, 18). However, up to 30% of patients do not present with classic symptoms and diagnosis can become more difficult (19–24). In these cases, doctors rely on clinical history, dermatological and neurological evaluation, and anamnesis (25). Recently, a few studies have used CT scans to characterize maxillary morphometrics for diagnosing and treating rhinomaxillary syndrome in contemporary patients with leprosy (26–29), hypothesizing that standardized anteroposterior measurements of the cranium and maxilla would be most useful to diagnose leprosy using maxillary morphology (specifically maxilla anteroposterior length divided by cranium anteroposterior length). This hypothesis was not supported (26–28).

This is important because delayed diagnosis amplifies the likelihood of bone changes and severe deformity is not uncommon in the hands, feet, and face of contemporary patients (30, 31). Characteristic facial deformities resulting from untreated leprosy [e.g., (11)] are responsible for stigmatization and prejudice against patients worldwide (32) and this can have severe consequences for their physical and mental health (33, 34). We provide three case studies that demonstrate the potential value of CT imaging for diagnosing leprosy in contemporary medical practice based on changes to the anterior maxilla, pyriform aperture, and bony palate, rather than the morphometrics used previously in clinical practice. Based on our analysis we argue that qualitative assessments using CT of morphological changes in the ventral maxilla and palate, visible in human skeletons of people with leprosy [e.g., (35)] provide a set of reliable diagnostic criteria for *in vitro* diagnosis in living humans for whom diagnosis is complicated or non-typical.

## 2 Materials and methods

Dahwa (56 4I’ 44.778″ E, 24″ 3′ 2.01” N) is an archaeological site located on a terrace at the eastern foothills of AI-Hajar Mountains. It is surrounded by Wadi al-Sarmi in the north, Wadi al-Shafan in the south, AI-Haiar Mountains in the west, and the coast of Batinah on the east. Its maximum elevation is 163 m above sea level. The Umm an-Nar period (2500–2000 BCE) sites (DH1, DH5, DH6, DH7, DH8) are located 24 km south-west of Saham and comprise of 61 buildings and two Umm an-Nar circular tombs (DH5-Tomb 1 and DH7-Tomb 1) (14–16). The full extent of the site is unknown as a road was constructed through the site. All necessary permits and permissions were obtained from the Ministry of Tourism and Heritage for the excavation and the osteological analysis.

The habitation and work areas of the site were excavated by the Department of Archaeology at Sultan Qaboos University (14, 16, 36). In 2013 and 2015 surveys were conducted to understand the extent of the site. In 2016, advanced geospatial methods were used to map the site (14, 16). The excavation of DH7 Tomb 1 and the associated bone pit was conducted by a team from Temple University from 2016–2019 and yielded more than 180,000 bone fragments and associated material culture, dated to 2500–2000 BCE. The context of the site is secure, as the chambers of this tomb had not been looted or disturbed by construction as many tombs from this period have experienced.

Dahwa is a first Umm an-Nar (UAN) period site excavated on the Batinah Coast, situated at a strategic location for trade between Mesopotamia and the Indus Valley civilization of South Asia. A large number of Indus pottery sherds (around 72% of the total sherds) indicate the first interaction of the Umm an-Nar people with the Indus people after 2500 BCE (36). There is also evidence of furnace linings and copper slag fragments demonstrating the extent of copper production at the site.

Like elsewhere across the Oman Peninsula, the UAN people of Dahwa interred their dead in finely built monumental stone tombs, which served as communal spaces for the dead and involved extensive secondary mortuary ritual(s) where the deceased were moved throughout the chambers of the tomb (when there is more than one chamber) and at some sites, into a subterranean bone pit as the final act of the ritual. Tomb1 at Dahwa was 8.2 m in diameter and consisted of 6 subterranean stone-walled chambers. Communication between the chambers was achieved with a central corridor. Ongoing research is exploring the mortuary ritual in this structure, but preliminary work supports the idea that the interred deceased were moved throughout the tomb over an unspecified period, eventually resting in the bone pit, several meters from the tomb itself. The interred material culture received the same treatment.

The mortuary ritual reduced the bones and material culture into more than one hundred thousand fragmented and commingled remains (more than 120,000 from the bone pit and 40,000 from the tomb). These were sorted, identified, counted, and weighed by Williams and Robbins Schug in 2018. The minimum number of individuals (MNI) interred in the tomb and the bone pit next to it was 200 people, estimated by Williams using a count of the left mastoid process. During this inventory process, a total of 69 maxillae and 56 mandible fragments were brought back to the University of North Carolina Greensboro for paleopathological analysis based on an initial observation of changes to the rhinomaxillary region. There were signs of antemortem tooth loss (AMTL) and alveolar resorption in the mandibles, traumatic injuries and chronic subperiosteal bone formation in long bone fragments, and *cribra orbitalia* (abnormal porosity) in eye orbits of frontal bone fragments. Given the nature of the commingled assemblage, these elements are not securely associated with one another and are not described here but will be subject for future analysis.

Rhinomaxillary syndrome has formed the basis for diagnosing leprosy in the cranial skeleton since it was first described, with the diagnostic utility of these specific bone lesions repeatedly confirmed (37–39). Osseous changes indicative of leprosy (in order of diagnostic value) include: (1) resorption of the anterior nasal spine that progresses from reduction of the length of spine to destruction of the cortical bone, exposure of the underlying medullary bone, and in some cases evidence for remodeling in the cortical bone; (2) antemortem resorption of the maxillary alveolar process between prosthion and subspinale; (3) destruction, including resorption and rounding, of the margins of the pyriform aperture, leading to the exposure of the nasal branch of the second trigeminal nerve (maxillary nerve V2); (4) inflammatory changes, pitting, localized destruction, periosteal new bone formation, and/or remodeling of the palate and interior of the pyriform aperture, including the nasal processes of the maxillae, nasal septum, inferior nasal conchae; and (5) evidence of periodontal inflammation and alveolar recession in the posterior dentition.

As a syndrome, these manifestations of rhinomaxillary disease occur in association but not every person with leprosy will express every lesion. They are all also potentially present in other chronic, inflammatory infectious diseases, such as treponemal infection and tuberculosis (TB). However, in skeletons where the first four of these skeletal changes are present together, this is considered to be pathognomonic for leprosy (40–42).

Conventional analyses of the dry bone included photographic documentation and visual observation of pathological changes to bones and teeth. In our evaluation of 69 maxillae from the Bone Pit and Tomb 1, we recorded the presence of dental caries, calculus (mineralized plaque deposits), and the lesions characteristic of leprosy described above. Twelve maxillae (8 from the Bone Pit and 4 from the Tomb) had four or more signs of severe rhinomaxillary syndrome, which suggested a probable diagnosis of leprosy (37–39). Nine of these maxillae were very fragmentary, consisting of only the ventral portion of the maxilla and palate. These were excluded from this analysis and will be subject to additional research on deep learning algorithms for diagnosis using micro-CT data in the future.

For this project, three adult maxillae were selected for micro-CT imaging (DH7 ID 012, 093, and 044). ID 012 and ID 093 consisted of right and left maxillae from the Bone Pit. ID 044 was a right and left maxilla from the Tomb. These three maxillae were chosen because both the right and left side were present; the two elements were either attached or were adjacent to one another in the tomb or in the bone pit; they had at least four signs of rhinomaxillary disease; and the relevant areas of the bone were well preserved for observation, without significant post-mortem damage. We also scanned one maxilla from the Biology Department Anatomy Collection at UNC Greensboro. This individual did not have any characteristic signs of disease and was scanned for reference.

We performed scans of four maxillae using a Nikon XT H 225 micro-CT scanner at the North Carolina Agricultural and Technical Institute (NC A&T) and University of North Carolina Greensboro (UNCG) Joint School of Nanoscience and Nanoengineering (JSNN) in Greensboro. The scanner was calibrated in August 2024. The archaeological samples are curated at UNCG’s main campus, in the Human Diversity Lab. To prevent movement in the micro-CT, the bones were placed in low density foam, inside of an in-house made Styrofoam sample holder. Dry bone specimens do not require further preparation (43). This was done prior to transporting them to JSNN to ensure the samples were protected during transportation to our satellite campus.

Detailed descriptions of how a micro-CT works, scan settings, and their effect on image quality have been published elsewhere (44). We used a standard scan protocol provided by Nikon and only relevant aspects are provided here. Samples were aligned with the maximum scan volume positioned perpendicular to the source and the detector. Voltage influences beam penetration into the sample, while amperage determines the brightness of the resulting radiograph. A scout scan was used to determine the scan parameters. X-ray source voltage was set between 150–156 kV, current ranged from 67–86 μA (specific values are provided in the figure caption for each scan). No filter was used for additional beam hardening minimization. Small rotation interval, high number of frame averaging, and longer scan times (75–90 min) were used to reduce the signal to noise ratio. To ensure adequate resolution to evaluate trabecular bone, voxel sizes were kept below 10 μm (values provided in the figure captions). The entire bone was included in the Field of View (FOV).

The image stacks were imported into Dragonfly™ to be reconstructed from the rotation image projections. Global thresholding was applied. We did not segment the images or apply algorithms to enhance image quality. No ROIs were created or quantification protocols were employed for this study. The reconstructed images were evaluated as 2D radiograph sections and in a 3D surface reconstruction. The surfaces of lesions, their internal tissue structure, presence of exposed trabeculae, and remodeled compact bone were evaluated. Image stacks, metadata, and reconstructions are stored on MorphoSource™, external hard drives, and backup drives at UNCG in the Human Diversity Lab. Scans are available on request.

## 3 Results

**Figure 1:**
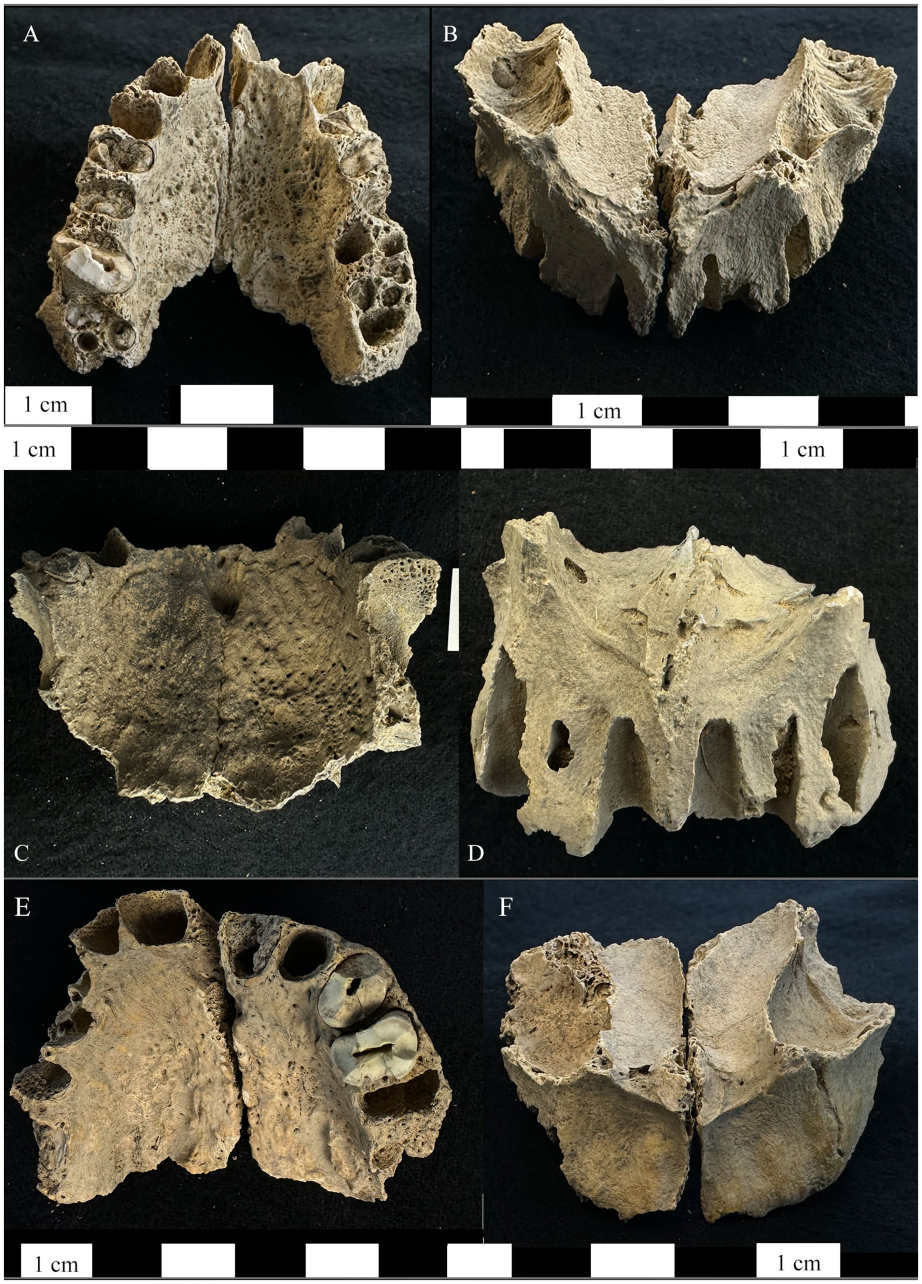
Right and left maxillae from the bone pit at Dahwa included in this study. **(A)** Maxilla ID 012 inferior-dorsal view of the maxilla demonstrating periosteal new bone formation and abnormal porosity on the palatal surface. **(B)** The superior-anterior view of ID 012 showing porosity on the cortical surface of the maxillary sinuses, exposure of maxillary nerve V2, resorption of the anterior nasal spine and the margins of the pyriform aperture, and alveolar resorption of the anterior dentition. **(C)** Maxilla ID 093 inferior-dorsal view of the maxilla demonstrating periosteal new bone formation and abnormal porosity on the palatal surface as well as abnormal porosity on the mesial alveolar wall for the left first molar. **(D)** The superior-anterior view of ID 093 showing resorption of the anterior nasal spine, margins of the pyriform aperture, alveolar resorption around the anterior dentition. **(E)** Maxilla ID 044 inferior-dorsal view of the maxilla demonstrating periosteal new bone formation and abnormal porosity on the palatal surface. **(F)** The superior-anterior view of ID 044 showing exposure of maxillary nerve V2, resorption of the anterior nasal spine, and alveolar resorption of the anterior dentition.

Our visual examination of maxillary bone deformity in the right and left maxillae from Dahwa ID 012 and 093 demonstrated four signs consistent with leprosy (Figure 1): periosteal new bone formation and evidence of inflammatory changes to the compact bone lining the palatal surface of the maxilla, the anterior nasal spine resorption (compact bone is intact and medullar bone is not exposed), progressive smooth resorption and recession of the margin of the pyriform aperture, and bilateral resorption of the alveolar process for ID 012 and 093 (8–12 mm for all alveoli of the anterior dentition while posterior teeth are unaffected). ID 044 has the first three signs above, moderate alveolar resporption, and two abscesses internal to the ventral maxilla. These signs provided strong diagnostic value however, it is difficult based solely on visual inspection to rule out taphonomic damage as the cause of some morphological changes, such as the changes to the anterior nasal spine, without radiological exam. Furthermore, making a diagnosis of leprosy in very fragmentary, disassociated, and commingled remains is challenging as the patterning of lesions across the skeleton cannot be observed. We used micro-CT as a non-invasive means to seek a deeper level of certainty in the diagnosis and to better characterize the microstructural aspects of lesions typically used to diagnose this disease in paleopathological analysis of fragmentary, commingled remains.

**Figure 2:**
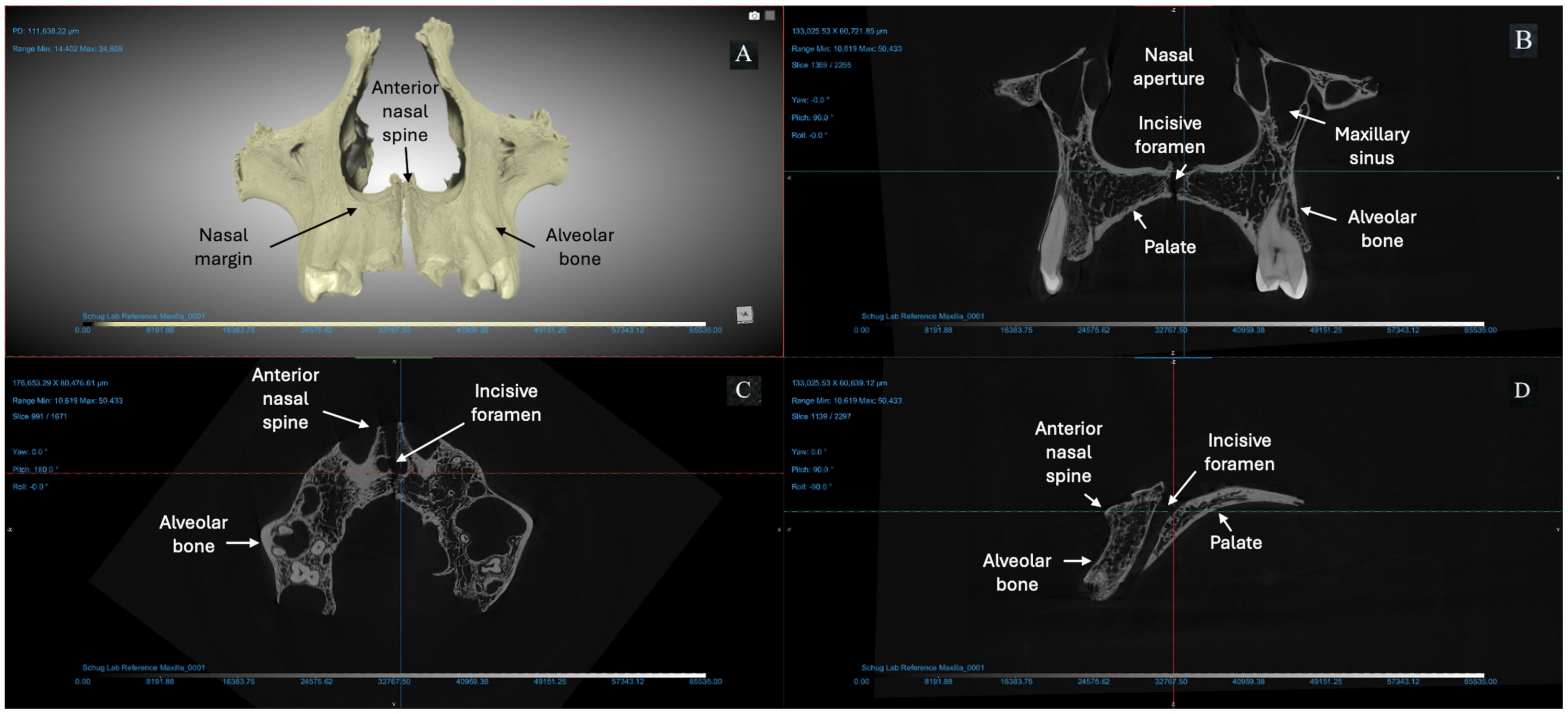
Micro-CT of right and left maxillae from a deceased individual from the anatomical reference collection at UNCG (156 KV, 76 μA, voxel size is 5.13 μm). **(A)** A 3D reconstruction of the bones from Dragonfly. The X-Z **(B)**, Y-Z **(C)**, and X-Y **(D)** views demonstrate typical morphology expected from an adult human maxilla: intact anterior nasal spine; sharp everted margin of the nasal aperture; mild to no alveolar resorption (< 2 mm) at anterior dentition; thin cortex of compact bone lining the pyriform aperture, maxillary alveoli, and palatal surface.

**Figure 3:**
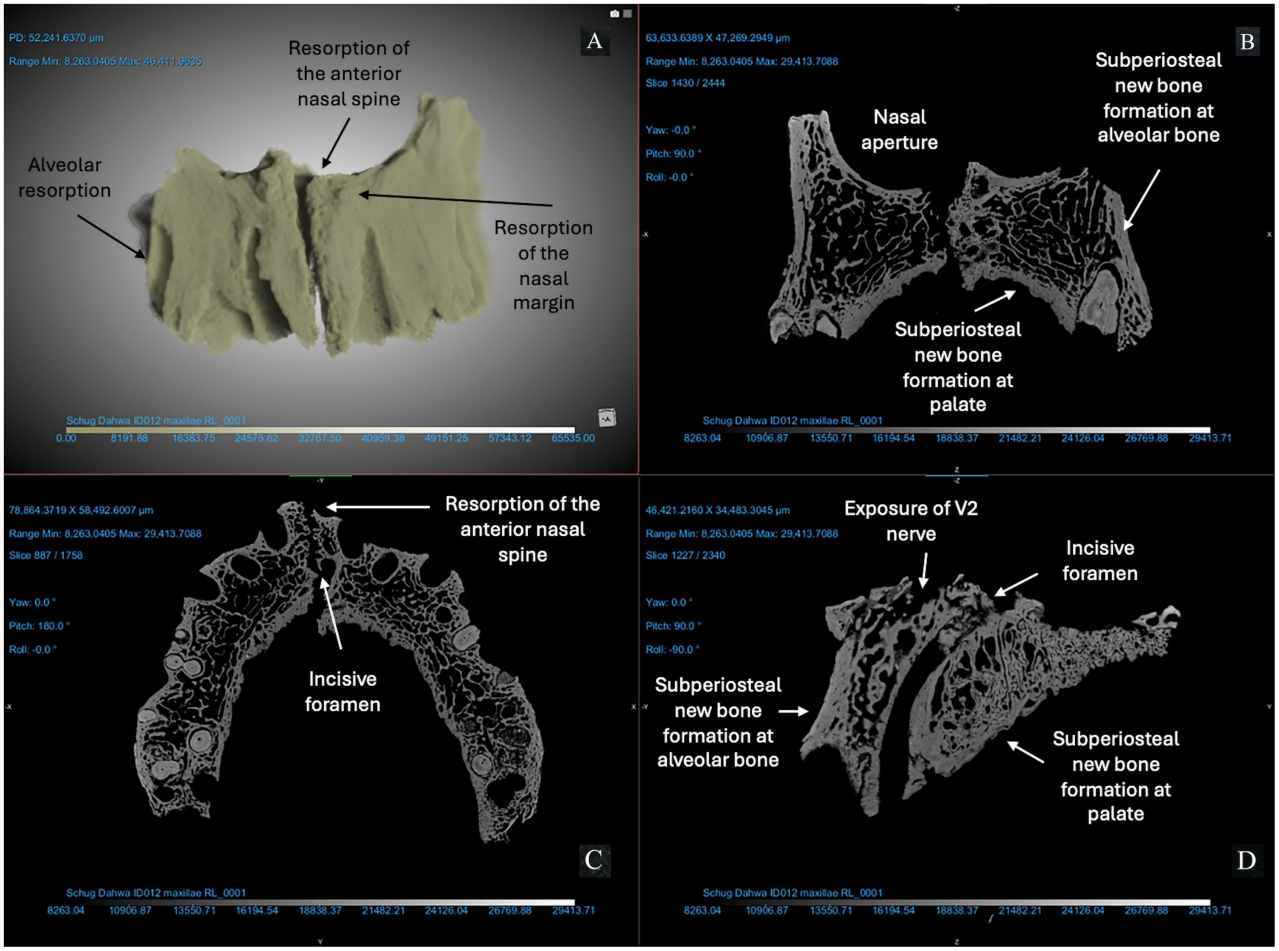
ID 012 micro-CT scan (150 KV, 67 μA, voxel size is 3.72 μm). Right and left maxillae **(A)** demonstrate significant changes to the anterior bone surfaces of the alveoli (right and left central incisors and canines), pyriform aperture, and palate. The X-Z **(B)**, Y-Z **(C)**, and X-Y **(D)** views demonstrate exposure of the V2 nerve and resorption of the anterior nasal spine; erosion of the margin of the nasal aperture; alveolar resorption (> 10 mm) at anterior dentition; subperiosteal new bone formation on the cortical bone lining the pyriform aperture and maxillary alveoli; and > 1 mm of bone proliferation on the palatal surface. Changes are marked with arrows in this figure.

**Figure 4:**
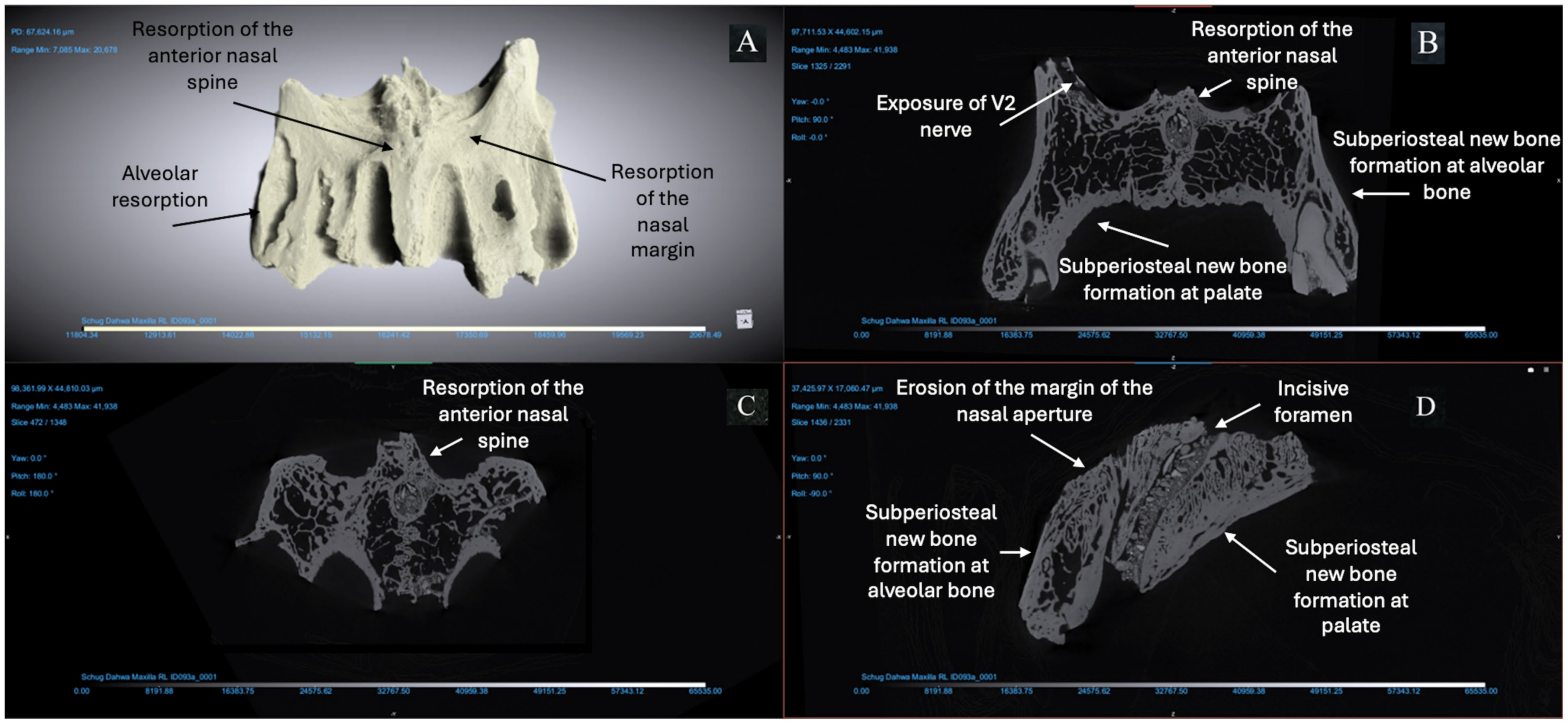
ID 093 micro-CT scan (150 KV, 81 μA, voxel size is 3.61 μm). Right and left maxillae **(A)** demonstrate significant changes to the bone surfaces of the anterior alveoli, pyriform aperture, and palate. The X-Z **(B)**, Y-Z **(C)**, and X-Y **(D)** views demonstrate resorption of the anterior nasal spine; erosion of the margin of the nasal aperture; alveolar resorption (> 10 mm) at anterior dentition; subperiosteal new bone formation on the cortical bone lining the pyriform aperture and maxillary alveoli; and > 1 mm of bone proliferation on the palatal surface.

**Figure 5:**
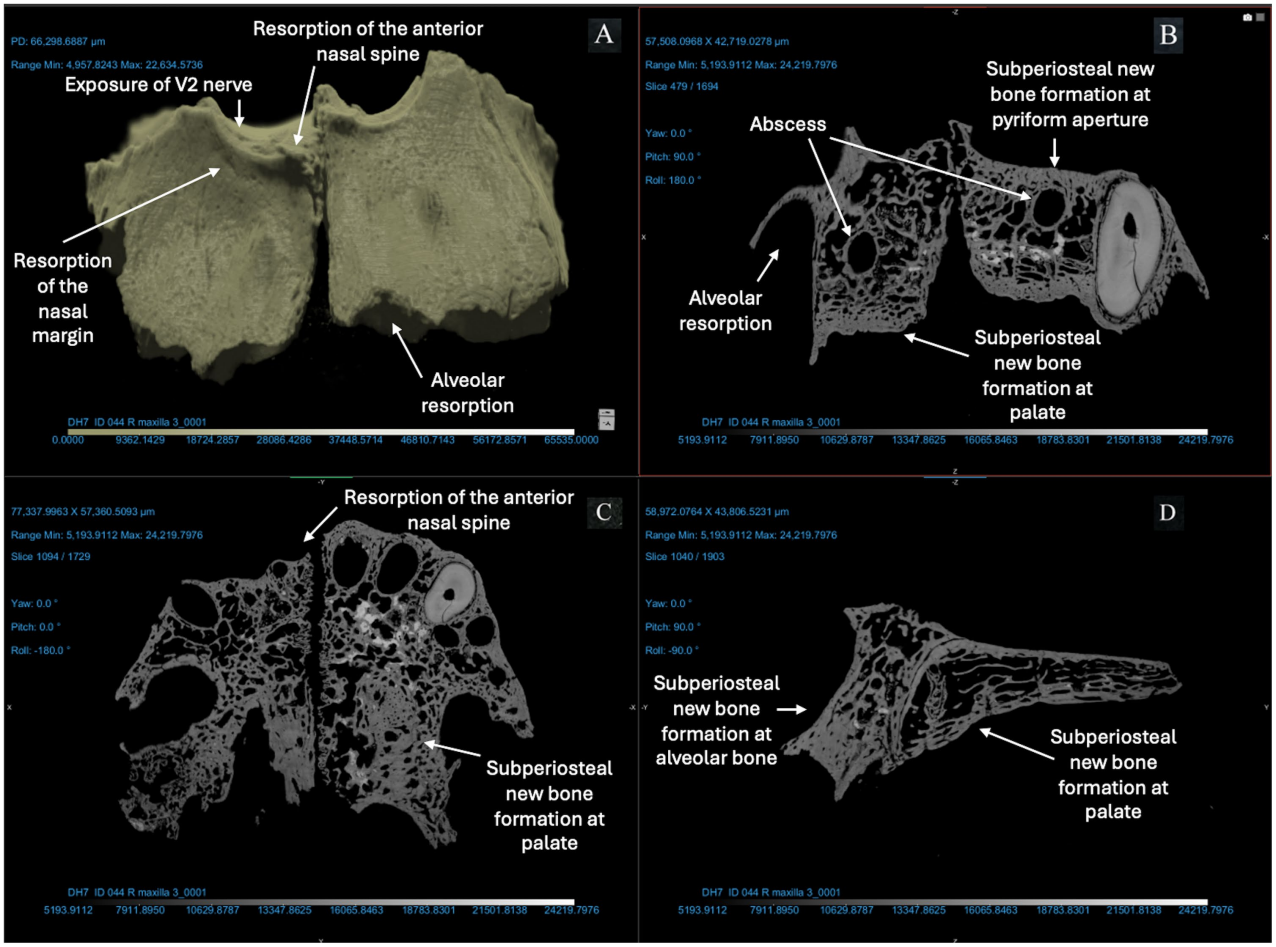
ID 044 micro-CT scan (150 KV, 86 μA, voxel size is 3.42 μm). Right and left maxillae **(A)** demonstrate significant changes to the bone surfaces of the anterior alveoli, pyriform aperture, and palate. The X-Z **(B)**, Y-Z **(C)**, and X-Y **(D)** views demonstrate resorption of the anterior nasal spine and exposure of the V2 nerve; alveolar resorption of 3–6 mm at right and left central incisors and canines; two abscesses of the right and left posterior alveoli (near the first molar); thickening of cortical bone lining the pyriform aperture and anterior maxillary alveoli; and > 1 mm of bone proliferation on the palatal surface.

We used micro-CT to evaluate a right and left maxilla from the anatomical collection of the UNCG Biology Department as a reference for typical morphology and tissue types (Figure 2). Compact bone lining the nasal and oral surfaces of this normal, reference maxilla is <1 mm in thickness. There is no evidence of periosteal new bone formation or abnormal porosity. The anterior nasal spine, nasal margin, and alveolar bone are intact (not resorbed or remodeled). We compared the morphology of this typical maxilla to micro-CT scans of three archaeological maxillae (Figures 3–5). In all cases, atrophy of the nasal spine, resorption of the anterior alveolar process, and loss of sharpness of the nasal margin were observed radiographically. We also observed evidence of chronic inflammation in the form of more than 1 mm of remodeled subperiosteal new bone formation on the palatal surface, a lesion resulting from longstanding inflammation in the palatal mucosa and contact with purulent lesions in the soft tissue.

## 4 Discussion

This paper has three major conclusions. First, we present a paleopathological analysis of archaeological bone from the site of Dahwa, Oman and provide the first evidence for morphological changes associated with lepromatous leprosy in Bronze Age Arabia. Second, we demonstrate morphological changes that characterize rhinomaxillary syndrome on a micro-structural level, which suggests the usefulness of this method for conducting non-invasive paleopathology analysis for fragmentary and commingled assemblages. Finally, this paper could have some value for contemporary clinicians using CT scanning to understand deformities resulting from undiagnosed leprosy.

This paper documents leprosy in Bronze Age Oman, at a near-coast settlement devoted to copper trade with Indus and other civilizations in the Middle Asian Interaction Sphere. Leprosy has previously been documented in India and Pakistan from the same time period (11, 12) but this is the first evidence of the pathogen outside of South Asia. Given the large number of Indus ceramics and iconography at Dahwa, it is clear that the site was involved in trade with Indus people; artifactual evidence suggests they were exporting copper across the Gulf and the Arabian Sea. The presence of leprosy in this population supports prior suggestions that pathogens moved along these trade routes as well (45, 46).

This paper suggests greater diagnostic certainty can be obtained using micro-CT in cases where leprosy is suspected in archaeological maxillae. In cases where whole skeletons are available and where lepromatous leprosy has led to severe disfigurement, visual examination of the skeleton is often sufficient [i.e., (11)]. However, there are many archaeological and historic sites where remains are disarticulated or commingled. This makes paleopathological diagnosis difficult because in those cases, maxillae cannot typically be associated with hand and foot bones or lower limb bones, which are also evaluated for differential diagnosis. This research demonstrates how micro-CT is valuable for diagnostic confirmation in ancient human skeletal remains because it allows changes such as subperiosteal new bone formation to be visualized using non-invasive methods, making it easier to rule out taphonomic damage and diagnose this disease with more certainty in a greater variety of burial communities.

Micro-CT is not available to all paleopathologists because they do not have the equipment, the funds, or the expertise to conduct the scans and analyze the data. In that case, collaboration is recommended to achieve greater diagnostic power. In addition, many researchers have questions as to whether micro-CT may limit the possibility for other types of analyses in the future, such as ancient DNA work. While there is no evidence of this in research conducted thus far [c.f., (47)], caution can be exercised through selecting samples for DNA work prior to micro-CT scanning.

This study may also have some value for contemporary clinicians. Leprosy is difficult to diagnose in 30% of contemporary patients due to difficulty culturing the pathogen *in vitro* or *in vivo* and many of these cases go undiagnosed (19–22, 24). Because delayed diagnosis puts these patients at risk of significant and permanent disfigurement, several studies have used CT scanning for diagnosis in contemporary patients (26–29). These studies used paleopathological literature to hypothesize that palatal anteroposterior length, divided by cranium anteroposterior length, would be most useful to diagnose leprosy. Given a closer reading of paleopathological literature [e.g., (11, 12, 35)] and experimental evaluation of contemporary leprosy patients, this hypothesis was not supported (26–28).

We suggest qualitative assessments of the ventral maxilla and palate would be more useful in CT scans of living patients. Our results suggest that clinicians should focus on qualitative assessments of the nasal aperture, palate, and the medial anterior portion of the maxilla, including: progressive resorption to total loss of the anterior nasal spine, resorption of the alveolar process that becomes crescentic (U-shaped), symmetrical loss of sharpness at the margins of the nasal aperture, reactive bone on the cortical surfaces of the maxilla, and evidence of chronic inflammation on the palate (periosteal new bone formation and resorption). While CT technology is expensive, radiographs would provide much less resolution on reactive bone changes and other lesions. CT is also indicated for those patients who are candidates for prosthetics.

## 5 Conclusion

More than 250,000 cases of leprosy are diagnosed each year, primarily in Global South countries, but some evidence suggests increasing incidence in regions where the disease is endemic (4). Due to the difficulties associated with culturing the pathogen, little is known about its origin, evolution, and early migrations. We demonstrate here that leprosy migrated to Oman and South Asia along trade routes for copper and other materials, which connected Afro-Eurasia in the Bronze Age. Our lab is working to understand the experience of Bronze Age connectivity and health using archaeological remains from the site of Dahwa. Because the human remains from this site, and other Arabian UAN sites, are highly fragmented and commingled, we are using micro-CT analysis, molecular, SEM-EDS, and genomics techniques to delve into the microstructural and microscopic evidence for the human experience. Future work in our lab will also use these micro-CT scans to test hypotheses about the specific etiology of the bone changes seen in rhinomaxillary syndrome.

This study adds to the limited number of studies evaluating rhinomaxillary changes using micro-CT in fragmentary and commingled assemblages. It also suggests that clinical characterizations of rhinomaxillary disease should focus on qualititative assessments of the ventral maxilla and demonstrates how paleopathology can provide solutions to diagnostic and other challenges in medicine through interdisciplinary collaboration that recognizes the different but complementary materials and expertise of paleopathologists and clinicians. It is well understood that paleopathology contributes to evolutionary medicine, which is indirectly important for clinical practice and public health (45). Our data can also be useful for diagnostics and for understanding the progression of some infectious diseases in the absence of medical intervention. This is particularly valuable for emerging and reemerging infections and those that are anti-biotic resistant.

## Data Availability

The raw data supporting the conclusions of this article will be made available by the authors, without undue reservation.
